# Rate, Timing, and Duration of Unplanned Readmissions Due to Cardiovascular Diseases among Hospitalized Patients with Cancer in the United States

**DOI:** 10.31083/j.rcm2411326

**Published:** 2023-11-23

**Authors:** Sola Han, Ted J. Sohn, Anton L.V. Avanceña, Chanhyun Park

**Affiliations:** ^1^Health Outcomes Division, College of Pharmacy, The University of Texas at Austin, Austin, TX 78712, USA

**Keywords:** readmission, cardiovascular disease, cancer, length of stay

## Abstract

**Background:**

Cardiovascular disease (CVD) can lead to unplanned care in patients with cancer, which may affect their prognosis and survival. We aimed to compare the rates, timing, and length of stay of unplanned CVD readmission in hospitalized patients with and without cancer.

**Methods:**

This study used the 2017–2018 Nationwide Readmissions Database to identify adult hospitalized patients with and without cancer. The primary outcome was 180-day unplanned CVD readmission rates. CVD was defined based on a composite variable that included atrial fibrillation, coronary artery disease, cardiomegaly, cardiomyopathy, heart failure, peripheral artery disease, and stroke. For patients readmitted due to CVD, the timing between admissions (based on the mean number of days between index hospitalization and readmission) and length of stay were further identified.

**Results:**

After matching, 300,398 patients were included in the two groups. The composite CVD readmission rates were significantly higher in patients with cancer (5.92% vs 4.10%; odds ratio (OR) 1.47, 95% CI 1.44–1.51, *p* < 0.001). Patients with cancer were also associated with shorter mean number of days to composite CVD readmission (60.48 days vs 68.32 days, *p* < 0.001) and longer length of stay of composite CVD readmission (8.21 days vs 7.13 days, *p* < 0.001). These trends were maintained in analyses of the individual CVD.

**Conclusions:**

Hospitalized patients with cancer experienced higher rates of unplanned readmission due to CVD, and their CVD readmissions occurred sooner and required longer lengths of stay compared to patients without cancer. Efforts to reduce unplanned CVD readmissions, such as providing optimized chronic post-discharge care, may improve the health outcomes of patients with cancer.

## 1. Introduction

As patients with cancer experience gains in life expectancy, the incidence of cardiovascular disease (CVD) in this population has also increased [1, 2]. CVD has been reported to be the most common cause of mortality in cancer survivors, and patients with all types of cancer have a higher risk of CVD-related death compared with the general population [3]. It has been known that cancer and CVD have overlapping risk factors (e.g., obesity, diabetes, or lower socioeconomic status) or similar underlying mechanisms (e.g., inflammation, or oxidative stress) [1, 2, 4]. In addition, there are increasing concerns about the cardiotoxicity of cancer therapies, such as radiotherapy and chemotherapies/immunotherapies, that can be associated with developing cardiovascular complications, including heart failure, coronary artery disease, cardiomyopathy, arrhythmia, peripheral artery disease, or stroke [2, 5, 6].

Incident CVD in patients with cancer may affect their risk of unplanned care such as readmissions [7], which has been shown to be associated with worse prognosis and survival [8]. Previous research estimates that 35% of patients with cancer experience an unplanned hospitalization within the first year after cancer diagnosis, of which 5.8% are due to cardiovascular reasons [9]. Although many unplanned readmissions may not be avoidable, studies have suggested that they can be reduced by timely and appropriate post-discharge care access and optimization of chronic care for patients with cancer [9, 10, 11, 12, 13].

Despite growing attention to CVD risk and unplanned readmissions in cancer patients, previous research has focused on limited types of CVD and cancer to estimate the incidence or prevalence of CVD [1, 14] or to evaluate CVD readmission rates [9, 15]. No study has evaluated the characteristics of readmissions (e.g., days to readmission and length of stay) across different CVD events and cancers. Studies have also used narrow time frames (e.g., 30 days) that do not fully capture the elevated CVD risk among patients with cancer [16]; previous research suggests that CVD risk among patients with cancer is higher than that of individuals without cancer from 6 months to over 10 years after diagnosis [17, 18]. The present study fills this research gap by evaluating the risk of unplanned 180-day CVD readmission among hospitalized patients with and without cancer. For patients readmitted due to CVD, we further evaluated the impact of cancer on the number of days to readmission and length of stay.

## 2. Materials and Methods

### 2.1 Data Source

We used the 2017 and 2018 Nationwide Readmissions Database (NRD) in this study. The NRD is a publicly available database of all-payer hospital inpatient stays and is part of the Healthcare Cost and Utilization Project (HCUP) that is sponsored by the Agency for Healthcare Research and Quality [19]. This database contains about 18 million discharges each year if unweighted, and about 35 million discharges in the United States if weighted [19].

### 2.2 Study Population

Patients were excluded from this study if they were (1) younger than 18 years old; (2) had any listed diagnosis of CVD (i.e., atrial fibrillation, coronary artery disease, heart failure, stroke, peripheral artery disease, cardiomegaly, and cardiomyopathy) in the index hospitalization; (3) had missing values in any baseline characteristics or length of stay; (4) discharged from July to December (as these hospitalizations would lack a minimum 180-day follow-up data); and (5) died during the index hospitalization.

### 2.3 Exposure

We divided the study population by their cancer status. Patients were classified as having cancer if they were admitted with a primary diagnosis of cancer (Clinical Classifications Software Refined categories of NEO001-NEO071) during the index hospitalization. Patients were classified as having no cancer if they were admitted without any listed cancer diagnosis.

### 2.4 Outcomes

The outcome of interest was 180-day unplanned CVD readmission rates between patients with and without cancer. The 180-day unplanned CVD readmissions were defined as the first CVD readmission within 180 days of discharge that was not elective. We defined a composite CVD readmission event based on the first occurrence of readmission for atrial fibrillation, coronary artery disease, cardiomegaly, cardiomyopathy, heart failure, peripheral artery disease, and stroke which we identified using International Classification of Disease, Tenth Revision, Clinical Modification (ICD-10-CM) codes (**Supplementary Table 1**) [20]. If a patient had multiple CVD readmissions within 180 days after the index hospitalization, only the first readmission was included in this study. For each CVD readmission, we further identified the (1) number of days between the index hospitalization and CVD readmission and (2) length of stay of CVD readmission.

### 2.5 Covariates

Baseline characteristics were obtained from the index hospitalization, including age, sex, components of the Elixhauser index for the risk of readmissions [21], household income, primary payer, hospital characteristics (i.e., bed size, ownership, and teaching status). Some components of the Elixhauser index that overlapped with cancer and CVD definitions of this study were excluded from the analysis.

### 2.6 Statistical Analysis

We used chi-square tests to assess differences in baseline characteristics and the proportion of patients with and without cancer who had a CVD readmission. We used *t*-tests to assess differences in the number of days to, and length of stay of, readmission between patients with and without cancer who were readmitted due to CVD. We performed propensity score matching, using a 1:1 matching approach with a caliper of 0.2 standard deviations (SD) of the logit of the propensity score. An absolute standardized difference of <0.1 was considered appropriate for achieving balance between the groups. To accommodate data preparation, environmental pertaining and analysis time and storage space, we used a 10% random sample of the non-cancer patients for propensity score matching. Logistic regression analyses were used to predict probabilities and odds ratio (OR) with 95% confidence interval (CI) of having CVD readmission. A *p*-value less than 0.05 was considered statistically significant. Before matching, all reported data were based on the weighted analyses to provide national estimates, corresponding to the NRD complex sampling design. After matching, we used unweighted cases for analyses. We performed statistical analyses using SAS version 9.4 (SAS Inc., Cary, NC, USA) and StataMP version 17 (StataCorp, College Station, TX, USA).

## 3. Results

### 3.1 Baseline Characteristics

**Fig. 1.**
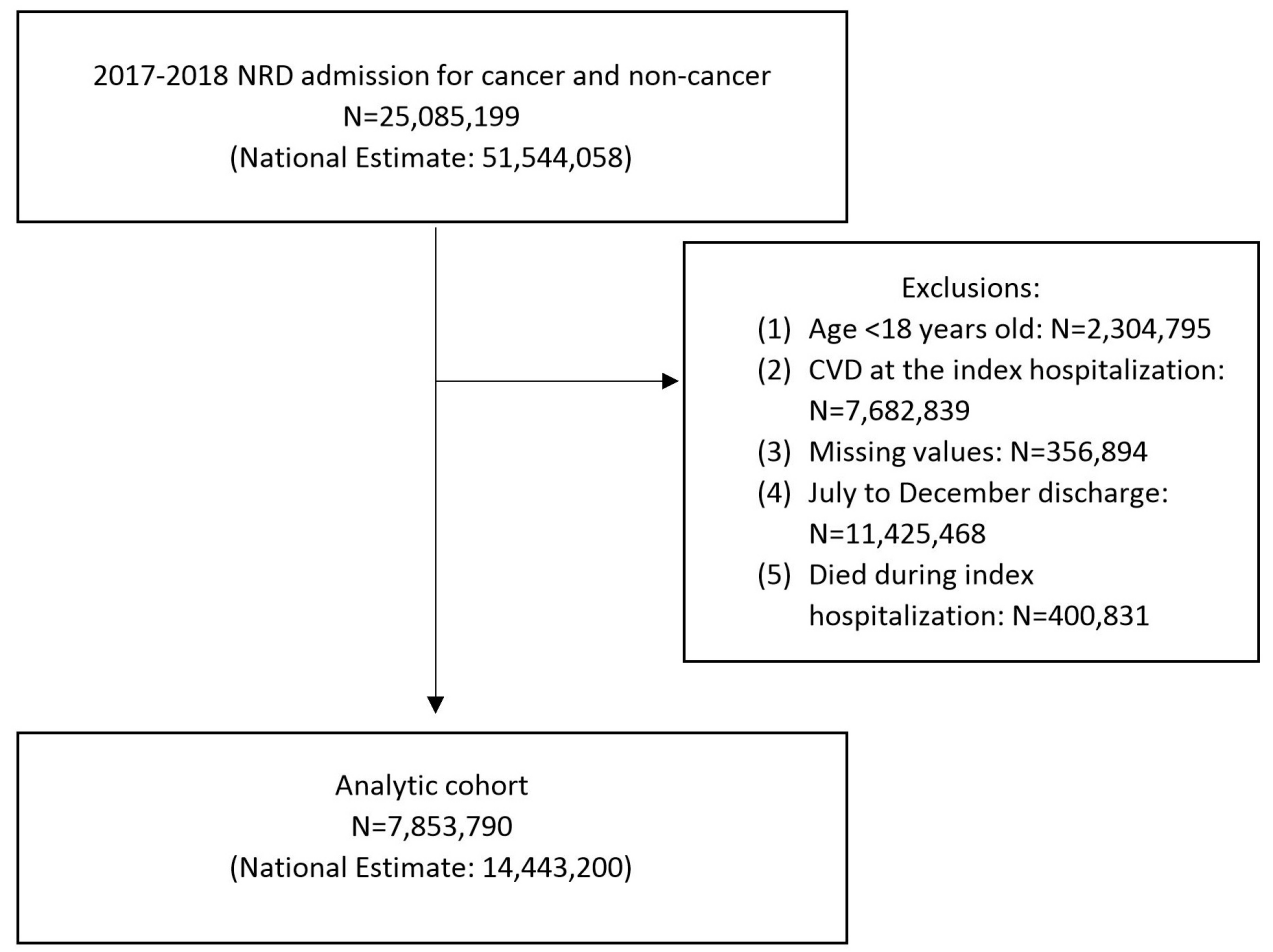
**Diagram flow of the study selection before propensity score matching**. CVD, cardiovascular disease; NRD, Nationwide Readmissions Database.

A total of 358,716 patients with cancer (national estimates: 640,623) and 7,495,074 patients without cancer (national estimates: 13,802,576) were included in this study (Fig. 1). Compared to the patients without cancer, patients with cancer had a higher prevalence of older age (40–59 years: 32.89% vs 25.76%; 60–79 years: 52.09% vs 24.21%; 80+ years: 8.74% vs 7.41%; *p* < 0.001), uncomplicated diabetes (11.52% vs 8.78%, *p* < 0.001), complicated hypertension (6.19% vs 5.85%, *p* < 0.001), uncomplicated hypertension (43.01% vs 29.95%, *p* < 0.001), chronic pulmonary disease (16.11% vs 14.06%, *p* < 0.001), hypothyroidism (10.85% vs 9.35%, *p* < 0.001), and other thyroid disorders (1.44% vs 0.91%, *p* < 0.001) at the time of index hospitalization (Table 1). After propensity score matching, a total of 300,398 patients with cancer and 300,398 patients without cancer were included, and all of patients’ baseline characteristics being assessed were balanced between the two groups (Table 2).

**Table 1.** **Baseline characteristics of study population before propensity score matching**.

		With cancer		Without cancer		*p-*value
		Unweighted N	358,716	Unweighted N	7,495,074	
		Weighted N	640,623	Weighted N	13,802,576	
		N	%	N	%	
Age groups						
	18–39	40,223	6.28	5,882,282	42.62	<0.001
	40–59	210,722	32.89	3,555,599	25.76	
	60–79	333,695	52.09	3,341,628	24.21	
	80+	55,984	8.74	1,023,068	7.41	
Sex						
	Male	313,969	49.01	4,465,855	32.36	<0.001
	Female	326,654	50.99	9,336,721	67.64	
Income						
	0–25th	161,480	25.21	4,117,461	29.83	<0.001
	26–50th	173,385	27.07	3,837,053	27.80	
	51–75th	161,172	25.16	3,314,737	24.02	
	76–100th	144,585	22.57	2,533,326	18.35	
Insurance						
	Medicare	288,118	44.97	4,124,076	29.88	<0.001
	Medicaid	73,892	11.53	3,372,187	24.43	
	Private insurance	246,633	38.50	5,062,685	36.68	
	Other	31,980	4.99	1,243,629	9.01	
Hospital bed size						
	Small	73,247	11.43	2,523,791	18.28	<0.001
	Medium	142,296	22.21	3,898,857	28.25	
	Large	425,080	66.35	7,379,928	53.47	
Hospital ownership						
	Government, nonfederal	75,238	11.74	1,585,556	11.49	<0.001
	Private, non-profit	507,705	79.25	10,244,278	74.22	
	Private, invest-own	57,680	9.00	1,972,742	14.29	
Hospital location and teaching						
	Metropolitan non-teaching	95,233	14.87	3,082,079	22.33	<0.001
	Metropolitan teaching	520,169	81.20	9,415,048	68.21	
	Non-metropolitan hospital	25,221	3.94	1,305,449	9.46	
Clinical conditions						
	Acquired immune deficiency syndrome	2740	0.43	68,974	0.50	<0.001
	Alcohol abuse	17,086	2.67	774,591	5.61	<0.001
	Autoimmune condition	12,932	2.02	340,613	2.47	<0.001
	Dementia	11,701	1.83	495,060	3.59	<0.001
	Depression	63,720	9.95	1,560,895	11.31	<0.001
	Diabetes with chronic complications	44,513	6.95	1,027,701	7.45	<0.001
	Diabetes without chronic complications	73,779	11.52	1,212,112	8.78	<0.001
	Drug abuse	9064	1.41	895,196	6.49	<0.001
	Hypertension, complicated	39,624	6.19	807,621	5.85	<0.001
	Hypertension, uncomplicated	275,526	43.01	4,134,177	29.95	<0.001
	Chronic pulmonary disease	103,199	16.11	1,940,739	14.06	<0.001
	Obesity	85,647	13.37	2,191,221	15.88	<0.001
	Hypothyroidism	69,503	10.85	1,289,961	9.35	<0.001
	Other thyroid disorders	9250	1.44	126,260	0.91	<0.001

**Table 2.** **Baseline characteristics of study population after propensity score matching**.

		With cancer		Without cancer	
		N	300,398	N	300,398
		N	%	N	%
Age groups					
	18–39	21,894	7.29	21,935	7.30
	40–59	106,886	35.58	108,101	35.99
	60–79	142,133	47.31	139,704	46.51
	80+	29,485	9.82	30,658	10.21
Sex					
	Male	132,629	44.15	133,752	44.52
	Female	167,769	55.85	166,646	55.48
Income					
	0–25th	74,626	24.84	75,588	25.16
	26–50th	78,958	26.28	79,418	26.44
	51–75th	76,227	25.38	76,280	25.39
	76–100th	70,587	23.5	69,112	23.01
Insurance					
	Medicare	136,030	45.28	136,527	45.45
	Medicaid	39,557	13.17	39,512	13.15
	Private insurance	108,643	36.17	107,609	35.82
	Other	16,168	5.38	16,750	5.58
Hospital bed size					
	Small	38,958	12.97	39,553	13.17
	Medium	74,891	24.93	75,862	25.25
	Large	186,549	62.1	184,983	61.58
Hospital ownership					
	Government, nonfederal	37,024	12.32	36,448	12.13
	Private, non-profit	230,706	76.8	230,343	76.68
	Private, invest-own	32,668	10.87	33,607	11.19
Hospital location and teaching					
	Metropolitan non-teaching	55,461	18.46	56,912	18.95
	Metropolitan teaching	233,772	77.82	231,980	77.22
	Non-metropolitan hospital	11,165	3.72	11,506	3.83
Clinical conditions					
	Acquired immune deficiency syndrome	1405	0.47	1404	0.47
	Alcohol abuse	9230	3.07	8790	2.93
	Autoimmune condition	6930	2.31	6774	2.26
	Dementia	6599	2.2	6612	2.2
	Depression	31,658	10.54	32,517	10.82
	Diabetes with chronic complications	23,410	7.79	23,798	7.92
	Diabetes without chronic complications	35,191	11.71	34,821	11.59
	Drug abuse	5178	1.72	4970	1.65
	Hypertension, complicated	19,930	6.63	20,249	6.74
	Hypertension, uncomplicated	130,021	43.28	132,331	44.05
	Chronic pulmonary disease	48,766	16.23	48,812	16.25
	Obesity	43,558	14.5	44,937	14.96
	Hypothyroidism	34,631	11.53	34,599	11.52
	Other thyroid disorders	4044	1.35	3946	1.31

All variables were balanced between the two groups (standardized difference <0.1).

### 3.2 Probability of CVD Readmission between Cancer and Non-Cancer Patients

**Fig. 2.**
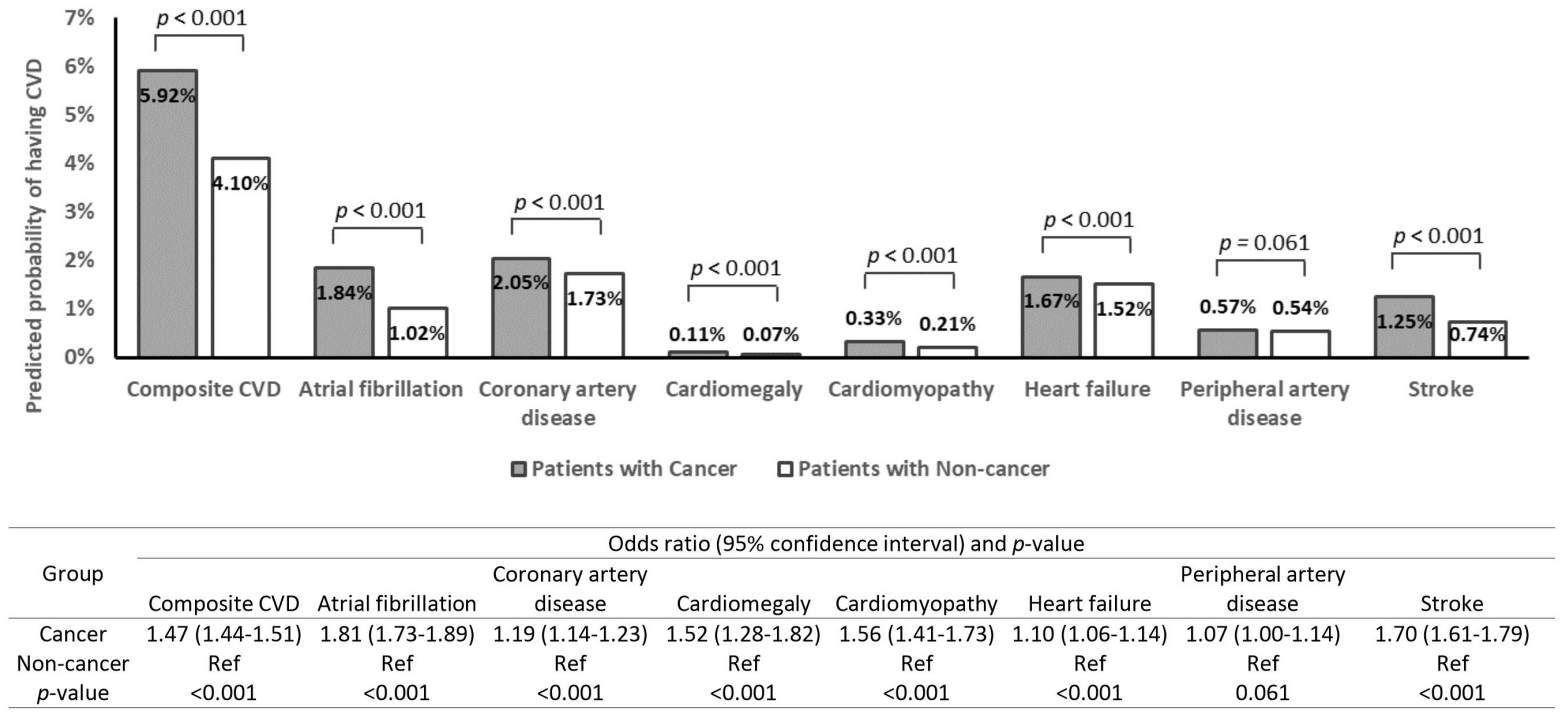
**Predicted percentages and odds ratio of having 180-day unplanned readmission due to cardiovascular diseases after propensity score matching**. CVD, cardiovascular disease.

After propensity score matching, the probabilities of having an unplanned 180-day readmission due to CVD are shown in Fig. 2. Patients with cancer had a higher probability of having readmission due to composite CVD (5.92% vs 4.10%; OR 1.47, 95% CI 1.44–1.51, *p* < 0.001), atrial fibrillation (1.84% vs 1.02%; OR 1.81, 95% CI 1.73–1.89, *p* < 0.001), coronary artery disease (2.05% vs 1.73%; OR 1.19, 95% CI 1.14–1.23, *p* < 0.001), cardiomegaly (0.11% vs 0.07%; OR 1.52, 95% CI 1.28–1.82, *p* < 0.001), cardiomyopathy (0.33% vs 0.21%; OR 1.56, 95% CI 1.41–1.73, *p* < 0.001), heart failure (1.67% vs 1.52%; OR 1.10, 95% CI 1.06–1.14, *p* < 0.001), peripheral artery disease (0.57% vs 0.54%, OR 1.07, 95% CI 1.00–1.14, *p* = 0.061), and stroke (1.25% vs 0.74%; OR 1.70, 95% CI 1.61–1.79, *p* < 0.001).

### 3.3 Number of Days to and Length of Stay of CVD Readmissions between Cancer and Non-Cancer patients

**Fig. 3.**
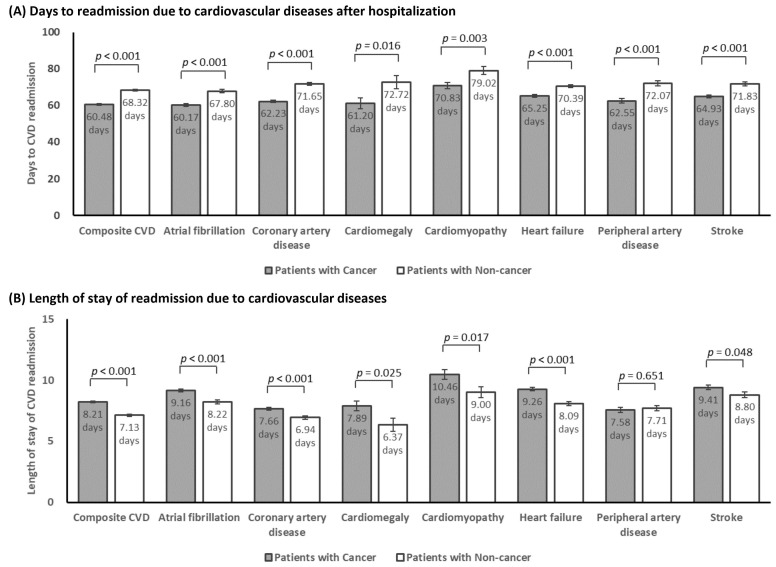
**Days to, and length of stay of, 180-day unplanned readmission due to cardiovascular diseases after propensity score matching**. (A) Days to readmission due to cardiovascular diseases after hospitalization. (B) Length of stay of readmission due to cardiovascular diseases. CVD, cardiovascular disease.

For those readmitted due to CVD within 180 days, the mean number of days to readmission was significantly shorter in patients with cancer compared to those without cancer (composite CVD: 60.48 days vs 68.32 days, *p* < 0.001; atrial fibrillation: 60.17 days vs 67.80 days, *p* < 0.001; coronary artery disease: 62.23 days vs 71.65 days, *p* < 0.001; cardiomegaly: 61.20 days vs 72.72 days, *p* = 0.016; cardiomyopathy: 70.83 days vs 79.02 days, *p* = 0.003; heart failure: 65.25 days vs 70.39 days, *p* < 0.001; peripheral artery disease: 62.55 days vs 72.07 days, *p* < 0.001; and stroke: 64.93 days vs 71.83 days, *p* < 0.001) (Fig. 3A).

In addition, significant differences were also found between patients with and without cancer on length of stay of CVD readmission. Patients with cancer were associated with significantly longer length of stay (composite CVD: 8.21 days vs 7.13 days, *p* < 0.001; atrial fibrillation: 9.16 days vs 8.22 days, *p* < 0.001; coronary artery disease: 7.66 days vs 6.94 days, *p* < 0.001; cardiomegaly: 7.89 days vs 6.37 days, *p* = 0.025; cardiomyopathy: 10.46 days vs 9.00 days, *p* = 0.017; heart failure: 9.26 days vs 8.09 days, *p* < 0.001; and stroke: 9.41 days vs 8.80 days, *p* = 0.048) except for peripheral artery disease (Fig. 3B).

## 4. Discussion

In this large population-based study, we found that patients with cancer experienced significantly higher risk for unplanned CVD readmissions that occurred sooner and led to a longer length of stay compared to patients without cancer. These trends were observed for each type of CVD we evaluated (i.e., atrial fibrillation, coronary artery disease, cardiomegaly, cardiomyopathy, heart failure, peripheral artery disease, and stroke). Considering that patients with cancer are at a higher risk of developing CVD, these findings provide valuable insights into understanding the impact of CVD on unplanned care in patients with cancer.

CVD is best evaluated using a relatively long follow-up time frame in cardio-oncology research [16], and our findings of 180-day unplanned CVD readmission rates provide new insights for evaluating CVD-related outcomes in patients with cancer. Compared to patients without cancer, the overall risk of coronary heart disease and stroke is higher among patients with cancer during the first 6 months through to 10 years after cancer diagnosis [17, 18]. Similar long-term trends have been reported for the risk of arrhythmia, heart failure, and venous thromboembolism among patients with cancer, compared to the general population [22]. In addition, it was reported that mean duration from immune checkpoint inhibitors and initiation to cardiovascular-immune-related adverse events ranged from 5–8 months [23].

Our observed general increase in CVD readmission rate in patients with cancer is similar to results from previous studies, although there are some differences in study design including study population definition, types of CVD, or readmission time frame [9, 15]. The causes of increased CVD readmission risk are likely to be varied including cardiotoxicity, which has been reported to be associated with many cancer-related therapies (e.g., radiotherapy, chemotherapy, or immunotherapy) [2, 5, 6], or relatively lower awareness or medical priority for CVD risk in patients with cancer [15, 16, 24]. However, as suggested by previous research, CVD risk may differ depending on cancer types, so further research is warranted. Nevertheless, our study results suggest that strategies to reduce CVD readmission for patients with cancer is needed to mitigate its negative effects on prognosis and mortality [15, 25].

In this study, we explored two aspects of readmission, namely timing and length of stay. The current analysis suggests that CVD-related readmissions can occur more quickly among previously hospitalized patients with cancer compared to those without cancer. To our knowledge, this study is the first to evaluate the potential impact of cancer on CVD readmission timing. In line with prior investigations, this study observed longer hospitalizations among readmitted patients with cancer [15]. The results of this study collectively highlight the need for transitional or post-discharge CVD preventive care for patients with cancer moving from an inpatient to an outpatient setting to reduce CVD-related unplanned readmissions. For example, the American Heart Association recommends a multimodal cardio-oncology rehabilitation model [26] that includes structured exercise training; nutritional counseling; weight, blood pressure, and diabetes management; tobacco cessation, and other interventions could reduce CVD risk among cancer survivors; however, the evidence around such programs are mixed [27]. Recently, the ERASE Trial in Canada reported that high-intensity interval training among patients with prostate cancer under active surveillance improved both cardiovascular and cancer outcomes [28]. To inform the design of future CVD prevention interventions, additional studies are needed to evaluate the modifiable predictors or causes of CVD readmissions in cancer patients.

### Study Limitations

First, due to the observational nature of our study design, although adjustment for demographics and comorbidities was made, there may still be residual confounders that underlie the observed association. The data used in this study contains only hospitalizations during a single year (no linkage is possible between years) and lacks information regarding cancer stages, prescribed drugs, laboratory data, race and ethnicity or other health care visit histories that do not result in hospitalization. Thus, caution is needed when interpreting the results. Second, due to the small number of CVD readmissions, we could not analyze CVD readmission risks stratified by cancer types, warranting further studies. Third, this study utilized the HCUP-NRD databases from 2017 to 2018 to mitigate the influence of COVID-19, as it falls beyond the scope of this study. In addition, the NRD includes data only from selected states, which may limit its generalizability to the entire population [19]. Fourth, similar to other databases, there is a possibility of missing or miscoding in the recorded causes of readmissions. As a result, this could have led to an overestimation or underestimation of the outcomes. However, the HCUP conducts regular quality control to ensure the validity and consistency of the data [29]. Fifth, our primary focus is on unplanned readmissions associated with CVD in the context of cardio-oncology. Consequently, future studies should consider exploring additional potential reasons that could contribute to readmissions among patients with cancer. Nevertheless, with a large sample size, this study provides a detailed overview of the risk, timing, and length of stay of CVD readmissions in patients with cancer which may be helpful for physicians and hospitals to better plan health care interventions for this population.

## 5. Conclusions

In this large population-based study of 600,796 patients with and without cancer, we found that patients hospitalized with cancer experienced a significantly higher risk of CVD readmission. In addition, patients with cancer tended to have CVD readmissions that occurred sooner and required longer hospital stays compared to patients without cancer, and these trends were identified across all individual CVD types (i.e., atrial fibrillation, coronary artery disease, cardiomegaly, cardiomyopathy, heart failure, peripheral artery disease, and stroke). These results suggest that efforts to reduce unplanned readmissions due to CVD by, for example, providing optimized chronic care and post-discharge care may be needed for patients with cancer.

## Data Availability

The data that support the findings of this study are available for purchase from the Central Distributor of the Healthcare Cost and Utilization Project (HCUP). To access the data, other researchers can contact HCUP through the HCUP Central Distributer (https://www.distributor.hcup-us.ahrq.gov) and purchasing the relevant years of HCUP data.

## Abbreviations

CI, confidence interval; CVD, cardiovascular disease; HCUP, healthcare cost and utilization project; ICD-10-CM, international classification of disease, tenth revision, clinical modification; NRD, nationwide readmissions database; OR, odds ratio.

## Supplementary Material

Supplementary material associated with this article can be found, in the online version, at https://doi.org/10.31083/j.rcm2411326.
