# Supplementary material for: Rate, Timing, and Duration of Unplanned Readmissions Due to Cardiovascular Diseases among Hospitalized Patients with Cancer in the United States
Source: Rev Cardiovasc Med. 2023 Nov 23;24(11):326. doi: 10.31083/j.rcm2411326 (PMC11272843; doi:10.31083/j.rcm2411326)
Supplement: Supplementary file 1 [file 2153-8174-24-11-326-s1.docx]

**Supplementary Online Content**

**Supplementary Table 1.** Diagnosis codes used to identify cardiovascular diseases

| **Cardiovascular disease types** | **ICD-10-CM diagnosis codes** |
| --- | --- |
| Atrial fibrillation | I48 |
| Coronary artery disease | I20-I25, I252 |
| Cardiomegaly | I517 |
| Cardiomyopathy | I43, I427, I429, I420, I425 |
| Heart failure | I50 |
| Peripheral artery disease | I70, I74, I739 |
| Stroke | I60-I63, I65-I66, I69, I672, I679, I6781-I6782 |
| Abbreviations: ICD-10-CM, International Classification of Diseases, Tenth Revision, Clinical Modification | |
